# Molecular screening to track ceftriaxone-resistant FC428-like *Neisseria gonorrhoeae* strains’ dissemination in four provinces of China, 2019 to 2021

**DOI:** 10.2807/1560-7917.ES.2025.30.6.2400166

**Published:** 2025-02-13

**Authors:** Leshan Xiu, Liqin Wang, Yamei Li, Lihua Hu, Jia Huang, Gang Yong, Youwei Wang, Wenling Cao, Yang Yang, Weiming Gu, Junping Peng

**Affiliations:** 1 NHC Key Laboratory of Systems Biology of Pathogens, National Institute of Pathogen Biology, Chinese Academy of Medical Sciences & Peking Union Medical College, Beijing, China; 2 School of Global Health, Chinese Center for Tropical Diseases Research, Shanghai Jiao Tong University School of Medicine, Shanghai, China; 3 One Health Center, Shanghai Jiao Tong University-The University of Edinburgh, Shanghai, China; 4 Key Laboratory of Respiratory Disease Pathogenomics, Chinese Academy of Medical Sciences and Peking Union Medical College, Beijing, China; 5 Department of Laboratory Medicine, The First Affiliated Hospital of USTC, Division of Life Sciences and Medicine, University of Science and Technology of China, Hefei, China; 6 Zhejiang Provincial Institute of Dermatology, Deqing, China; 7 Department of Laboratory Medicine and Sichuan Provincial Key Laboratory for Human Disease Gene Study, Sichuan Provincial People's Hospital, University of Electronic Science and Technology of China, Chengdu, China; 8 Guangzhou Institute of Dermatology, Guangzhou, China; 9 Shanghai Skin Disease Hospital, Tongji University School of Medicine, Shanghai, China; 10 Key Laboratory of Pathogen Infection Prevention and Control (Peking Union Medical College), Ministry of Education, Beijing, China; 11 State Key Laboratory of Respiratory Health and Multimorbidity, Chinese Academy of Medical Sciences, Beijing, China; * These authors contributed equally to this work.

**Keywords:** *Neisseria gonorrhoeae*, Antimicrobial resistance, A311V mutation, Molecular screening, FC428 clone, Epidemiological surveillance

## Abstract

**Background:**

The global dissemination of ceftriaxone-resistant *Neisseria gonorrhoeae* FC428-like strains poses a public health concern. To assess and follow their spread, establishing effective antimicrobial resistance (AMR) surveillance systems is essential.

**Aim:**

This study aimed to track ceftriaxone-resistant FC428-like strains in parts of China, using a molecular screening tool.

**Methods:**

Samples were collected from Sichuan, Zhejiang, Shanghai, and Guangdong provinces between 2019 and 2021. We employed a rapid molecular tool − the high-resolution melting analysis-based FC428 (HRM-FC428) assay, to screen for FC428-like strains. All FC428-like strains detected were further characterised by genotyping and PCR-sequencing.

**Results:**

Of 1,042 tested samples, 44 harboured the *penA*-60.001 allele linked to ceftriaxone resistance, revealing a 4.2% prevalence of FC428-like strains. The HRM-FC428 assay additionally uncovered six strains with mosaic *penA*-195.001 or *penA*-232.001 alleles, both bearing the A311V mutation, a ceftriaxone resistance marker. During the study, the prevalence of FC428-like strains among overall samples appeared to increase, with rates of 2.8% (11/395) in 2019, 4.2% (16/378) in 2020, and 6.3% (17/269) in 2021. Some strains’ sequence types (ST)s were identified across provinces (e.g. ST1903, ST1600) and most strains (24/44) were ST1903, an ST also reported in other regions/countries, suggesting local evolution and global transmission.

**Conclusion:**

Our work underscores the value of culture-independent antimicrobial resistance monitoring and validates the use of molecular diagnostic tools, like the HRM-FC428 assay for this purpose. This study offers insights into the complex landscape of ceftriaxone-resistant *N. gonorrhoeae*, emphasising the importance of continued surveillance and global collaboration to mitigate this growing public health threat.

**Key public health message**

**What did you want to address in this study and why?**

The rise of ceftriaxone-resistant gonococcal strains, particularly FC428-like variants, poses a considerable public health concern. We employed a molecular diagnostic tool to screen clinical samples for FC428-like strains in four Chinese provinces between 2019 and 2021. We aimed to understand the extent and implication of these strains’ dissemination as well as their diversity to inform surveillance and control strategies.

**What have we learnt from this study?**

Among 1,042 patient samples with *Neisseria gonorrhoeae* in the four provinces, 44 (4.2%) bore FC428-like strains. During the study, the prevalence of FC428-like strains among samples appeared to increase each year. Strains had diverse genetic sequences, with some sequence types identified across provinces, including one prior reported internationally. This suggested their local evolution and global transmission.

**What are the implications of your findings for public health?**

Because the spread of ceftriaxone-resistant FC428-like strains can challenge future use of ceftriaxone as first-line therapy, our findings emphasise the need for enhanced antimicrobial resistance (AMR) surveillance in *N. gonorrhoeae*. For this, we advocate the use of molecular diagnostic tools such as HRM-FC428. Global collaboration is also key to develop effective strategies to mitigate AMR in sexually transmitted pathogens.

## Introduction

*Neisseria gonorrhoeae* infections affect populations on a global scale, according to a report from the World Health Organization (WHO), with an estimated 82.4 million new cases worldwide for the year 2020 [1]. While reliable national case report data are not available everywhere, increases in gonorrhea case rates have recently been observed in many countries [2]. Meanwhile, the development of antimicrobial resistance (AMR) in this pathogen has narrowed therapeutic options for affected patients, leaving ceftriaxone monotherapy or in combination with azithromycin as the first and last line treatment for uncomplicated gonorrhoea [3]. Further exacerbating the problem has been the emergence and widespread dissemination of gonococcal FC428-like strains [4], which, as the initially reported FC428 clone, have the *penA*-60.001 allele with the crucial A311V mutation contributing to ceftriaxone resistance [5,6]. When additionally presenting with azithromycin resistance [5,6], these ceftriaxone-resistant strains lead to considerable therapeutic challenges [7]. Therefore, establishing effective surveillance systems in the world to prioritise and facilitate screening and tracking of FC428-like strains constitutes a necessary measure to support the containment of their sustained global dissemination, as well as to inform treatment guidelines [5,8,9].

Currently, several methods are available to identify and screen for FC428-like strains. These methods rely on both phenotypic and genotypic approaches, which primarily involve the isolation of *N. gonorrhoeae*, followed by antimicrobial susceptibility testing (AST) and subsequent genotyping through gene-based or whole genome sequencing [10,11]. Their complexity and long turnaround times, however, represent practical limitations for timely surveillance. To address these limitations, molecular diagnostic tools targeting specific genetic markers have been developed. These tools include real-time quantitative PCR [12,13], nucleic acid spectrometry [14], nanopore sequencing [15,16], and the high-resolution melting analysis-based FC428 (HRM-FC428) assay [17], which is the focus of this study. The assay offers a rapid, cost-effective, and efficient method for detecting FC428-like strains directly from clinical isolates or swabs [17], with previous studies having successfully applied it to detect FC428-like strains in clinical samples collected in the Shenzhen [17] and Changsha [6,18] cities of China, shedding light on the prevalence of clinically significant resistant clone lineages.

Given the increasing importance of FC428-like strains, it is key to gain information on their population structure and the intricate interplay between evolution and genetic diversity underlying their AMR. While the understanding of the worldwide diversity of these strains and the outbreaks that they have caused within individual countries has improved, more information on the origins and dissemination of FC428-like strains across different regions at national levels remains to be gained. To address this critical knowledge gap, we employed the HRM-FC428 assay to analyse clinical samples collected from several provinces across China over a 3-year period (2019−2021). With this study, we aim on one hand to further assess the potential of HRM-FC428 for surveillance of FC428-like strains, and on the other, to obtain insights on the dynamic spread of these strains to guide targeted public health interventions.

## Methods

### Study design and specimens

The research and collection of samples are part of an ongoing surveillance aimed at monitoring ceftriaxone-resistant FC428-like *N. gonorrhoeae* strains in China. In a multicentre approach, two sorts of samples were collected from four distinct hospitals across four provinces in China during the period of 2019−2021 as illustrated in Supplementary Figure S1. The hospitals participating in this study are the largest hospitals in their respective provinces and serve the entire provincial population. A total of 773 *N. gonorrhoeae* isolates (725 male; 48 female) were collected from clinical departments of the four participating hospitals between 2019 and 2020. These isolates were derived from individuals diagnosed with symptomatic gonorrhoea or urethritis, characterised by symptoms such as dysuria and/or urethral discharge. Each patient was represented by a single isolate in this collection. Additionally, 718 urogenital swabs (473 male; 245 female) were collected from consecutive outpatients suspected of either gonorrhoea or urethritis in 2021. Details on the sample inclusion are presented in the Supplementary Materials and Methods.

### Specimen processing and antimicrobial susceptibility testing

For 773 *N. gonorrhoeae* isolates, the nucleic acid extraction was performed post-revival on gonococcal medium supplemented with 1% IsoVitaleX (Oxoid, United States) [19]. The minimum inhibitory concentration (MIC) test was performed for each isolate adhering to the stipulated guidelines of Clinical and Laboratory Standards Institute (CLSI) [20]. The MIC value was determined according to the clinical breakpoints recommended by the European Committee on Antimicrobial Susceptibility Testing (EUCAST) [21].

Regarding the 718 urogenital swabs, Dacron swabs were procured from each patient and transported to the laboratory under ambient conditions (approximately 25°C). A portion of the eluate from each swab was directly used for nucleic acid extraction and screened for FC428-like strains using the HRM-FC428 assay, which we found in our previous study to effectively identify *N. gonorrhoeae* infections by targeting both *porA* and *opa* genes [17]. Swab samples that tested positive for *N. gonorrhoeae* underwent further processing for bacterial isolation and AST as described above [20,21].

### Evaluating the applicability of A311V mutation screening using HRM-FC428 assay

In our previous study [17], we found the HRM-FC428 assay was capable of detecting FC428-like strains as well as other strains or samples containing the A311V mutation in the *penA* gene. To further evaluate its applicability for screening for FC428-like strains in samples (nucleic acid extracts from either urogenital swabs or isolates) collected from four provinces, as well as the feasibility of A311V mutation screening, the HRM-FC428 assay was performed following the procedures outlined in the Supplementary Methods.

### Genetic characterisation

*N. gonorrhoeae* multiantigen sequence typing (NG-MAST), multilocus sequence typing (MLST), and *N. gonorrhoeae* sequence typing for antimicrobial resistance (NG-STAR) were performed for all FC428-like strains (NG-MAST, http://www.ng-mast.net; MLST, http://pubmlst.org/neisseria/; NG-STAR, https://ngstar.canada.ca), according to previous studies [17,18]. Full-length *penA* sequencing was performed as stated in Supplementary Methods to further validate all FC428-like strains discovered by the HRM-FC428 assay. A total of 44 *N. gonorrhoeae* FC428-like strains found in this work were analysed in context of prevalent NG-STAR-STs or MLST-STs reported in previous studies using the goeBURST algorithm [16]. Full-length nucleotide or derived amino acid sequences of mosaic* penA* alleles identified in study samples, as well as publicly available mosaic *penA* alleles associated with ceftriaxone resistance from *N. gonorrhoeae* and commensal *Neisseria* spp. were aligned and maximum-likelihood-based phylogenetic analyses were conducted with MEGA X.

### Statistical analysis

The parameters of specificity, sensitivity, positive predictive value (PPV), negative predictive value (NPV) and 95% confidence intervals (CI)s were calculated for the HRM-FC428 assay relative to the reference method (PCR-sequencing) using the VassarStats website (http://www.vassarstats.net/).

## Results

### Prevalence of A311V mutation and FC428-like strains in the samples

**Figure 1:**
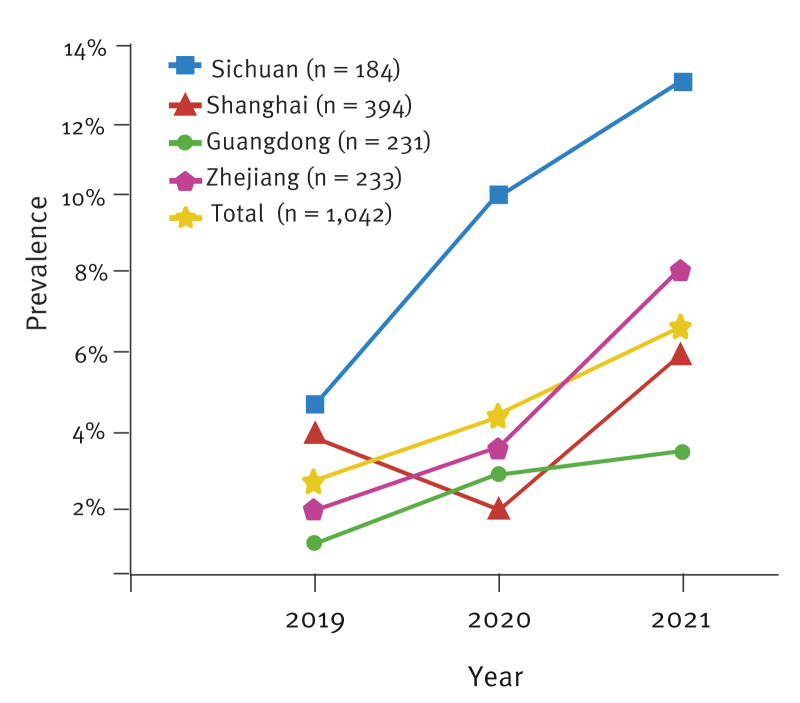
Prevalence over time of *Neisseria gonorrhoea *FC428-like strains among samples collected from four provinces, China, 2019−2021 (n = 1,042 samples)

Our assay revealed that among all samples collected in the study, a total of 1,042 samples (69.89% of 1,491) tested positive for *N. gonorrhoeae*, comprising 395 isolates (100% of 395) collected in 2019, 378 isolates (100% of 378) collected in 2020, and 269 swabs (37.47% of 718) collected in 2021. Among these 1,042 samples, 50 (4.80%) were found to contain the A311V mutation through HRM-FC428 testing. When PCR-sequencing was used to confirm this, 44 of the 50 samples (4.22% of 1,042) from 41 male and three female patients were classified as *penA-*60.001, indicating ceftriaxone resistance associated with the internationally spreading gonococcal FC428 clone. Additionally, in silico analysis using the NG-STAR online database with PCR-sequencing data characterised the remaining six samples as mosaic *penA* alleles containing A311V, including two *penA-*195.001 and four *penA-*232.001 [22,23]. At the province level, the prevalence of FC428-like strains was 7.61% (14/184) in Sichuan, which appeared to be higher than that in Shanghai (4.31%, 17/394), Zhejiang (3.43%, 8/233), and Guangdong (2.16%, 5/231) (Table 1, Figure 1). Among the samples, the prevalence of FC428-like strains seemed as if it was continuously increasing during the period 2019−2021, with rates of 2.78% (11/395) in 2019, 4.23% (16/378) in 2020, and 6.32% (17/269) in 2021 (Table 1, Figure 1). Similarly, the proportion of FC428-like strains in each of the four participating provinces seemed to steadily increase during the study period, rising between 2019 and 2021 from 4.35% to 13.64% in Sichuan, from 3.96% to 5.76% in Shanghai, from 1.96% to 8% in Zhejiang, and from 1% to 3.23% in Guangdong (Figure 1, Table 1).

**Table 1:** Percentage of *Neisseria gonorrhoeae* FC428-like strains in samples from four distinct provinces, China, 2019–2021 (n = 1,042 samples)

Year	Prevalence rate of FC428-like strains														
	Sichuan			Zhejiang			Shanghai			Guangdong			All four provinces		
	Number	Total	%	Number	Total	%	Number	Total	%	Number	Total	%	Number	Total	%
2019	4	92	4.35	2	102	1.96	4	101	3.96	1	100	1	11	395	2.78
2020	7	70	10	4	106	3.77	2	102	1.96	3	100	3	16	378	4.23
2021	3	22	13.64	2	25	8	11	191	5.76	1	31	3.23	17	269	6.32
2019−21	14	184	7.61	8	233	3.43	17	394	4.31	5	231	2.16	44	1,042	4.22

### Genotyping of the FC428-like strains identified in this study

**Figure 2:**
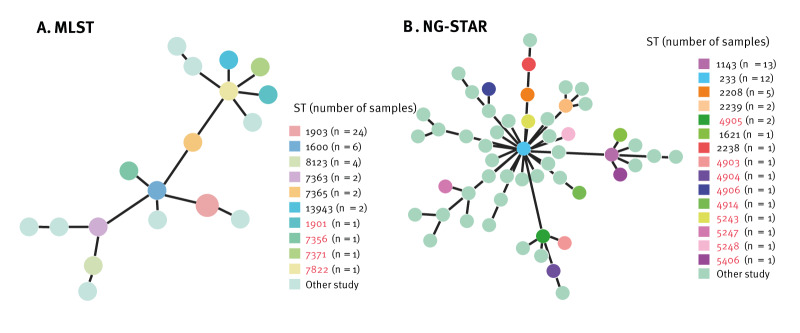
Genotyping of 44 FC428-like strains identified in this study, China, 2019–2021

Genotyping results of the 44 FC428-like strains using NG-STAR, MLST and NG-MAST are summarised in Table 2. Strains from different provinces exhibited diverse sequence types (STs), suggesting that these FC428-like strains may have evolved independently in different regions, with the *penA*-60.001 allele being present in various STs across these areas. MLST analysis of the 44 samples classified the FC428-like strains into 10 MLST-STs (Figure 2A), with MLST-ST1903 being the most predominant type (24/44), followed by MLST-ST1600 (6/44), MLST-ST8123 (4/44), MLST-ST13943 (2/44), MLST-ST7363 (2/44), and MLST-ST7365 (2/44). In contrast, global reports have identified over 200 FC428-like strains, which were categorised into 21 MLST-STs [24]. The lineages found within the collection from the provinces may have multiple sources. Some of the STs detected in our collection, were however not previously associated with FC428-like strains (Figure 2A), such as ST1901, ST7356, ST7371, and ST7822; these STs are known to be linked with resistance to multiple antibiotics worldwide [25].

**Table 2:** Characteristics of patients and samples associated with the FC428-like strains containing *penA-*60.001 allele, China, 2019–2021 (n = 44 samples)

**Sample name**	**Sex^a^**	**Clinical diagnosis**	**Type**	**Province**	**MIC, mg/L**			**Sequence type**		
					**CRO^b^**	**CFM^c^**	**AZM^d^**	**MLST**	**NG-STAR**	**NG-MAST**
CD19-21	Male	Gonorrhoea	Isolate	Sichuan	0.5	0.25	0.5	1903	233	3435
CD19-46	Male	Gonorrhoea	Isolate	Sichuan	0.5	0.25	0.125	1903	1143	22261
CD19-81	Male	Gonorrhoea	Isolate	Sichuan	0.5	0.25	0.25	8123	4903	22262
CD19-97	Male	Gonorrhoea	Isolate	Sichuan	0.5	0.25	0.5	1903	1143	22261
CD20-6	Male	Gonorrhoea	Isolate	Sichuan	0.5	0.125	0.5	1903	1143	22261
CD20-7	Male	Gonorrhoea	Isolate	Sichuan	0.5	0.125	0.25	7356	4906	22265
CD20-37	Male	Gonorrhoea	Isolate	Sichuan	0.25	0.125	0.0625	8123	4904	22266
CD20-48	Male	Gonorrhoea	Isolate	Sichuan	0.5	0.125	0.25	1903	1143	22261
CD20-59	Male	Gonorrhoea	Isolate	Sichuan	0.0156	0.0625	0.125	7822	5406	NA
CD20-60	Male	Gonorrhoea	Isolate	Sichuan	0.5	0.125	0.25	1903	1143	22261
CD20-64	Male	Gonorrhoea	Isolate	Sichuan	0.5	0.125	0.5	8123	4905	22269
CD21-4	Male	Urethritis	Swab	Sichuan	0.125	0.5	0.5	8123	4905	22175
CD21-17	Female	Urethritis	Swab	Sichuan	0.25	0.125	≥ 1	1903	233	NA
CD21-27	Male	Urethritis	Swab	Sichuan	0.25	0.5	≥ 1	7365	1143	22270
GZ19-42	Male	Gonorrhoea	Isolate	Guangdong	0.5	1	0.06	1903	5243	22274
GZ20-07	Male	Gonorrhoea	Isolate	Guangdong	1	> 1	0.125	1600	2208	22273
GZ20-51	Male	Gonorrhoea	Isolate	Guangdong	0.5	> 1	0.125	1903	1143	22261
GZ20-91	Male	Gonorrhoea	Isolate	Guangdong	1	> 1	0.125	1903	1143	22261
GZ21-10	Male	Gonorrhoea	Swab	Guangdong	0.016	1	0.03	1600	5247	1754
SH19-34	Male	Gonorrhoea	Isolate	Shanghai	0.5	2	0.06	1903	1143	22275
SH19-72	Male	Gonorrhoea	Isolate	Shanghai	0.5	2	0.06	1600	2208	22276
SH19-79	Male	Gonorrhoea	Isolate	Shanghai	0.5	2	0.125	1903	2239	NA
SH19-101	Male	Gonorrhoea	Isolate	Shanghai	0.5	2	0.125	1903	4914	22277
SH20-95	Male	Gonorrhoea	Isolate	Shanghai	0.5	2	0.125	13943	233	21711
SH20-167	Male	Gonorrhoea	Isolate	Shanghai	0.5	> 2	0.06	1903	1143	22261
SH21-6	Male	Gonorrhoea	Swab	Shanghai	NA	NA	NA	1903	233	22279
SH21-69	Male	Urethritis	Swab	Shanghai	0.03	0.06	1	1903	233	NA
SH21-76	Male	Gonorrhoea	Swab	Shanghai	NA	NA	NA	13943	233	21711
SH21-145	Male	Urethritis	Swab	Shanghai	0.03	0.25	4	1903	1143	22261
SH21-186	Male	Urethritis	Swab	Shanghai	0.5	0.25	0.5	1901	233	12108
SH21-197	Male	Urethritis	Swab	Shanghai	NA	NA	NA	1903	233	3435
SH21-232	Male	Gonorrhoea	Swab	Shanghai	0.5	2	4	7365	1621	22281
SH21-295	Male	Urethritis	Swab	Shanghai	0.5	2	0.5	1903	233	21711
SH21-296	Male	Urethritis	Swab	Shanghai	NA	NA	NA	7371	5248	22282
SH21-358	Female	Urethritis	Swab	Shanghai	0.25	2	0.5	1903	233	NA
SH21-366	Male	Gonorrhoea	Swab	Shanghai	0.5	2	8	1903	1143	NA
ZJ19-F1	Male	Gonorrhoea	Isolate	Zhejiang	0.25	2	0.125	1600	233	22284
ZJ19-F9	Male	Gonorrhoea	Isolate	Zhejiang	0.25	1	0.25	1903	1143	22285
ZJ20-23	Male	Gonorrhoea	Isolate	Zhejiang	0.5	1	0.5	1903	2239	22286
ZJ20-230	Male	Gonorrhoea	Isolate	Zhejiang	1	2	2	1600	2238	16059
ZJ20-231	Male	Gonorrhoea	Isolate	Zhejiang	1	2	2	1600	2208	22287
ZJ20-215	Male	Gonorrhoea	Isolate	Zhejiang	1	> 2	0.25	1903	233	21711
ZJ21-test7	Male	Gonorrhoea	Isolate	Zhejiang	1	2	> 4	7363	2208	22283
ZJ21-test12	Female	Gonorrhoea	Isolate	Zhejiang	1	2	> 4	7363	2208	22283

AZM: azithromycin; CFM: cefixime; CRO: ceftriaxone; EUCAST: European Committee on Antimicrobial Susceptibility Testing; MIC: minimum inhibitory concentration; MLST: multilocus sequence typing; NA: not available; NG-MAST: *Neisseria gonorrhoeae* multiantigen sequence typing; NG-STAR: *N. gonorrhoeae* sequence typing for antimicrobial resistance.

^a^ Sex was collected as a binary variable representing biological sex at birth (male or female).

^b^ Resistance to CRO was defined as a MIC ≥ 0.125 mg/L according to the EUCAST clinical-resistance breakpoint [21].

^c^ Resistance to CFM was defined as a MIC ≥ 0.125 mg/L according to the EUCAST clinical-resistance breakpoint [21].

^d^ Resistance to AZM was considered as a MIC > 1 mg/L according to the EUCAST’s epidemiological cut-off (ECOFF) in absence of EUCAST clinical-resistance breakpoint [21].

MLST: multilocus sequence typing;* N. gonorrhoeae*: *Neisseria gonorrhoeae* NG-STAR: *N. gonorrhoeae* sequence typing for antimicrobial resistance; ST: sequence type.

A. Each MLST-ST for* N. gonorrhoeae* FC428-like strains, which was identified in the current study, is represented in a distinct colour, corresponding to its name in the legend. The MLST-STs for FC428 and FC428-like strains found in previous global studies, but not in the current study, are indicated by a unique separate colour, corresponding to ‘Other study’ in the legend. In the legend, the name of MLST-STs not previously associated with FC428-like strains are highlighted in red. Publicly available MLST-STs sequences were downloaded from the MLST database (http://pubmlst.org/neisseria/).

B. Each NG-STAR-ST for *N. gonorrhoeae* FC428-like strains, which was identified in the current study, is represented in a distinct colour, corresponding to its name in the legend. Prevalent NG-STAR-STs for FC428 and FC428-like strains in previous global studies, but not detected in the current study, are indicated by a unique separate colour, corresponding to ‘Other study’ in the legend. In the legend, NG-STAR-STs not previously associated with FC428-like strains are highlighted in red. Publicly available NG-STAR-STs sequences were downloaded from the NG-STAR database (https://ngstar.canada.ca).

NG-STAR analysis identified 15 unique STs (Figure 2B), with the five most prevalent STs being NG-STAR-ST1143 (13/44), NG-STAR-ST233 (12/44), NG-STAR-ST2208 (5/44), NG-STAR-ST2239 (2/44), and NG-STAR-ST4905 (2/44). The clustering analysis revealed that the FC428-like strains from the current study distribute on multiple branches of the tree of global STs of FC428 and FC428-like strains, however, some clades on this tree mainly include strains found in samples from other studies (Figure 2B).

Among the 44 *N. gonorrhoeae* FC428-like strains identified by the HRM-FC428 assay, 38 samples were typed with NG-MAST and distributed among 25 NG-MAST-STs, while the remaining six samples could not be typed due to uninformative sequences. The most abundant ST among the 38 samples was NG-MAST-ST22261, found in nine samples, followed by NG-MAST-ST21711 (4/38), NG-MAST-ST22283 (2/38), and NG-MAST-ST3435 (2/38) (Table 2). Additionally, 21 of the 25 NG-MAST-STs were represented by only one sample.

### Accuracy evaluation of the HRM-FC428 assay in screening A311V mutation and FC428-like strains

**Figure 3:**
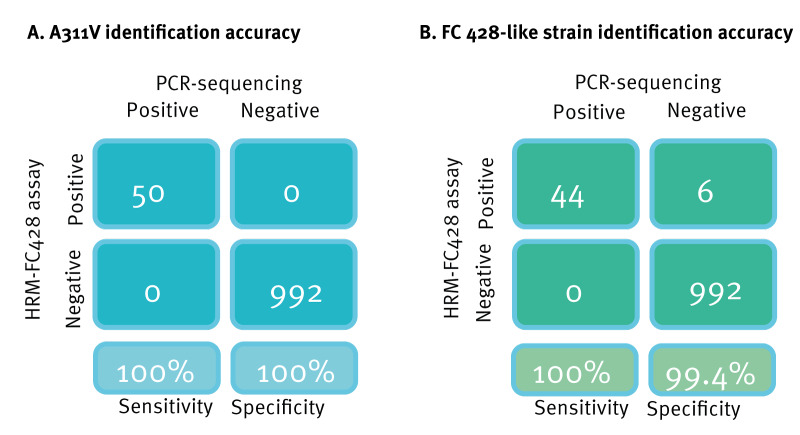
Accuracy evaluation of the HRM-FC428 assay in identifying (A) the A311V mutation and (B) FC428-like strains, China, 2019–2021 (n = 1,042 samples)

All 50 positive samples were further confirmed as mosaic *penA* allele with the A311V mutation through *penA* sequencing. The 100% agreement between the HRM-FC428 and PCR-sequencing method for identifying the A311V alteration demonstrates the robustness of the HRM-FC428 assay in detecting this crucial resistance marker (Figure 3A). Compared with PCR-sequencing analysis, the HRM-FC428 assay showed 100% sensitivity and 99.40% specificity in detecting FC428-like strains with the *penA-*60.001 allele in clinical samples (Figure 3B).

A. Evaluation of HRM-FC428 assay’s sensitivity and specificity for identifying the A311V mutation compared to standard PCR-sequencing.

B. Evaluation of HRM-FC428 assay’s sensitivity and specificity for detecting FC428-like strains compared to standard PCR-sequencing.

## Characterisation of the mosaic *penA* alleles with A311V mutation

**Figure 4:**
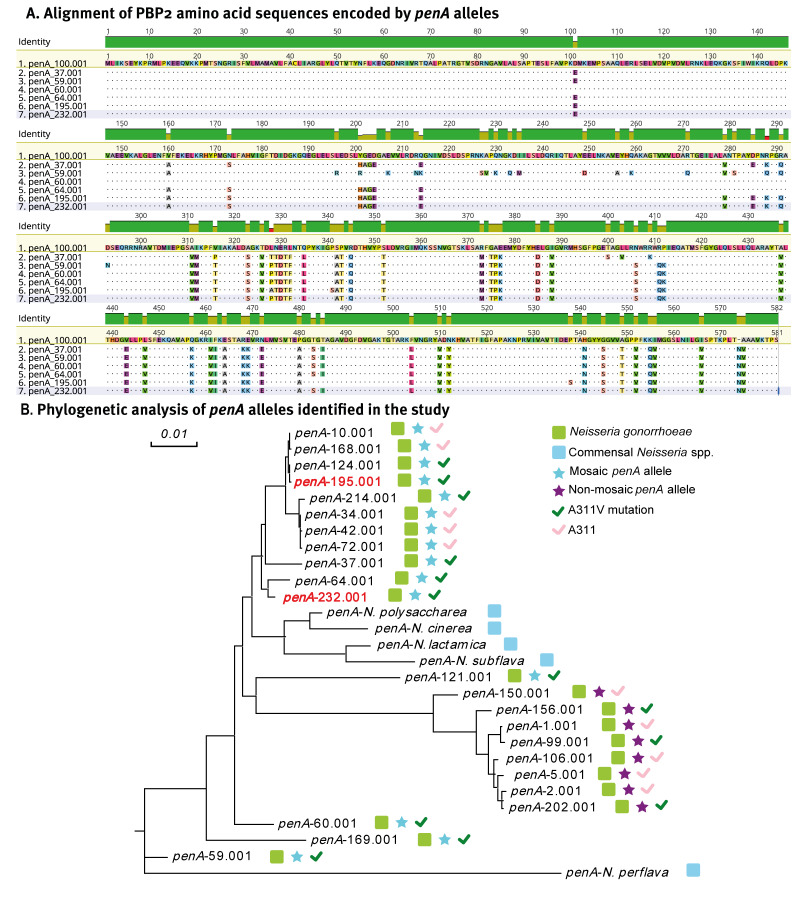
Characterisation and phylogenetic analysis of two mosaic *penA* alleles with the A311V mutation, which were detected in the study, China, 2019–2021

The six strains identified by HRM-FC428 testing as having the A311V mutation, but with a *penA *subsequently not characterised *as penA*-60.001, exhibited resistance to ceftriaxone or cefixime in AST, further underlining the risk of the evolution and spread of extended-spectrum cephalosporin (ESC)-resistant strains. Phenotypic and molecular characteristics of the six ceftriaxone-resistant *N. gonorrhoeae* strains with the mosaic *penA* alleles are shown in Supplementary Table S1. To identify the genetic relationship between these strains’ mosaic *penA* alleles, whose sequences are shown in Supplementary Table S2, and other mosaic *penA* alleles associated with ceftriaxone resistance, we performed both nucleotide and amino acid sequence alignments. Full-length *penA* sequencing of the mosaic *penA*-195.001 and *penA-*232.001 detected using the HRM-FC428 assay showed 95.83%, and 96.97% homology with the mosaic allele *penA-*60.001 of the FC428-like strains, respectively. The *penA-*195.001 encoded a mosaic penicillin-binding protein 2 (PBP2) with specific amino acid substitutions (A311V, I312M, V316T, N513Y and G545S) associated with ESC resistance (Figure 4A). On the other hand, the *penA-*232.001 nucleotide sequence shared 98.74% identical to that observed in *penA-*64.001 (A8806 strain [26] identified in Australia in late 2013) and contained key ceftriaxone resistance mutations, namely A311V, I312M, V316T, T483S, N513Y and G545S (Figure 4A). To further investigate the relevance of the mosaic *penA* alleles with previously reported sequences, the PBP2 amino acid sequences determined this study were subjected to phylogenetic analysis (Figure 4B). Phylogenetic analysis indicated that the mosaic *penA* alleles (*penA*-195.001 and *penA*-232.001) cluster with other mosaic *penA* alleles harbouring the A311V mutation. These alleles are carried by ceftriaxone-resistant strains.

PBP2: penicillin-binding protein 2.

A. Alignment of the amino acid sequences of the *penA* alleles encoding PBP2.

B. Phylogenetic analysis of *penA* full-length sequences identified this study and from previously reported *Neisseria gonorrhoeae* strains and commensal *Neisseria* spp. The mosaic *penA* alleles determined this study are highlighted in red. Publicly available *penA* sequences were downloaded from the NG-STAR database (https://ngstar.canada.ca) and the GenBank database (https://www.ncbi.nlm.nih.gov/genbank/).

The scale distance corresponds to the number of substitutions per site.

## Discussion

The emergence and global dissemination of ceftriaxone-resistant FC428 clones pose notable challenges to gonorrhoea management and control efforts worldwide [7,27]. This study employed a molecular diagnostic tool, HRM-FC428, to screen for FC428-like strains in patient samples collected from four Chinese provinces between 2019 and 2021. We observed a considerable presence of FC428-like strains in samples from all areas investigated, with Sichuan (7.61%) appearing to have a higher prevalence than Shanghai (4.31%), Zhejiang (3.43%), and Guangdong (2.16%). Moreover, over the study period, the prevalences found each year in each of the provinces seemed to increase. Given the existing gaps in *N. gonorrhoeae* AMR surveillance across different parts of China, the epidemiology of FC428-like strains at whole country-level remains incompletely understood. Our findings, however, together with those of others [8,28,29], suggest that FC428-like strains are not limited to specific geographical areas but are more widely dispersed nationally.

Typing as part of the current work further revealed a diverse landscape of MLST, NG-STAR and NG-MAST STs. In MLST genotyping, most domestic FC428-like strains belonged to MLST-ST1903, consistent with results from previous investigations in China and other countries [28,30-34], which suggests the existence of global dissemination networks. Moreover, we identified several MLSTs, such as MLST-ST1901, MLST-ST7356, and ST7822, which were previously known to be associated with resistance to multiple antibiotics [22,28,29,35], including ceftriaxone. The presence of *penA-*60.001 in strains of these STs may further enhance their ceftriaxone resistance ability.

Acquisition of *pen*A-60.001 could lead to the emergence of new clones at the local level, followed by the erosion of signals of clonality through recombination, and in some identifiable cases, the production of new clonal clusters. Strains in such clusters could in turn form the basis of new lineages capable of spreading globally over relatively short timeframes. In our study, genotyping by NG-STAR and NG-MAST further emphasised the diversity of FC428-like strains, highlighting the dynamic nature of their populations and their ability to rapidly adapt and spread.

Overall, the results of genotyping suggest direct or indirect transmissions of FC428-like strains within provinces in China and even over geographically distant areas, indicating that some of them may be part of nationally circulating lineages. Across the four provinces that we investigated, spread of some FC428-like ESC-resistant strains also seems to have occurred. While the patterns and prevalences of resistant genotypes that we found in the four studied provinces might possibly be indicative of the situation in other Chinese regions, comprehensive nationwide surveillance data are needed to confirm this.

Our study additionally highlights the robustness of the HRM-FC428 assay to accurately detect strains carrying the A311V mutation in the *penA* gene, a critical marker for ceftriaxone resistance [28,36]. The work moreover shows the successful implementation of this assay to detect infections with FC428-like strains bearing the *penA-*60.001 allele. This suggests that it can be a reliable tool for routine screening and surveillance of such strains in clinical settings, to facilitate early warning of their dissemination as well as efforts against their spread. Being applicable not only to clinical isolates but also to swabs [17], the assay supports ways to enhance culture-independent surveillance for ceftriaxone-resistant markers directly on clinical specimens, in line with WHO recommendations [37]. With its relative rapidity [17], the HRM-FC428 assay may potentially be suitable for upscaling and broader adoption in the future.

In the current investigation, the HRM-FC428 assay could additionally distinguish diverse *penA* alleles, in strains harbouring A311V mutation, such as *penA-*195.001 and *penA-*232.001, which share significant homology with the FC428 clone-associated *penA-*60.001 mosaic allele. Importantly, both mosaic *penA* alleles exhibit key resistance mutations implicated in ESC resistance, underscoring their clinical relevance and potential impact on treatment efficacy. The mutations in these alleles highlight the ongoing evolution and diversification of ceftriaxone-resistant strains, which warrants tracking their transmission and closely assessing their impact on treatment outcomes. Further research and monitoring are crucial to stay ahead of the evolving ceftriaxone-resistant landscape in *N. gonorrhoeae*.

Despite the promising results, our study has several limitations. Firstly, samples in 2019–2020 and 2021 were respectively collected using different approaches and the overall sample size is relatively small so may not fully represent the diversity of *N. gonorrhoeae* strains across different regions in China. Secondly, while the HRM-FC428 assay demonstrated high specificity and sensitivity in detecting the A311V mutation in the *penA* gene, there are inherent limitations associated with primer design and specificity, which may affect the assay's performance in detecting strains with decreased susceptibility. Moreover, the HRM-FC428 assay focuses on a single marker, and other genetic factors such as mutations in *mtrR*, *ponA*, and *porB* genes, which also contribute to ceftriaxone resistance, were not assessed in this study. Future research should aim to incorporate additional genetic markers to provide a more comprehensive approach to AMR surveillance and to better understand the mechanisms contributing to resistance in *N. gonorrhoeae.* Lastly, while this study emphasises the potential of the HRM-FC428 assay for early detection and monitoring of ceftriaxone-resistant strains, further validation in larger and more diverse populations is necessary to confirm its effectiveness and utility in various epidemiological settings.

## Conclusion

Our study contributes insights into the understanding of FC428-like strains in the Chinese context, shedding light on their prevalence and genetic diversity across the surveyed provinces. The widespread distribution of these strains and their ability to acquire additional resistance mechanisms emphasise the need for measures to preserve effective treatment options for gonorrhoea. Enhanced AMR surveillance constitutes one of such measures and the HRM-FC428 assay holds potential as a practical tool for targeted screening, monitoring, and early warning of ceftriaxone-resistant strains. Additionally, implementing targeted interventions to control the spread of FC428-like strains, such as partner notification and treatment, screening programmes in high-prevalence settings (e.g. sexually transmitted infection clinics), and pre-exposure prophylaxis for populations with a high risk of becoming infected, is essential. Furthermore, fostering collaboration between countries and regions to share data, resources, and best practices for FC428-like strains’ surveillance and control is of high importance.

## Supplementary Data

437000 Supplementary Material
