# Supplementary material for: Molecular screening to track ceftriaxone-resistant FC428-like Neisseria gonorrhoeae strains’ dissemination in four provinces of China, 2019 to 2021
Source: Euro Surveill. 2025 Feb 13;30(6):2400166. doi: 10.2807/1560-7917.ES.2025.30.6.2400166 (PMC11914965; doi:10.2807/1560-7917.ES.2025.30.6.2400166)
Supplement: Supplementary Material [file 2400166_SupplementaryMaterial.pdf]

This supplementary material is hosted by Eurosurveillance as supporting information alongside the article [Molecular screening to track ceftriaxone-resistant FC428-like *Neisseria gonorrhoeae* strains dissemination in some provinces of China, 2019 to 2021], on behalf of the authors, who remain responsible for the accuracy and appropriateness of the content. The same standards for ethics, copyright, attributions and permissions as for the article apply. Supplements are not edited by Eurosurveillance and the journal is not responsible for the maintenance of any links or email addresses provided therein.

Supplementary Materials

Supplementary Materials and Methods

Neisseria gonorrhoeae isolates and clinical samples

Hospital outpatient clinics serve as the primary point of initial contact for sexually transmitted infections (STIs) in China. Individuals can voluntarily seek care at local specialized dermatology hospitals or dermatology departments within comprehensive hospitals, regardless of whether they exhibit clinical symptoms or simply wish to undergo screening for STIs.

Two sorts of samples were taken for this cross-sectional research from hospitals in different geographic regions of China: Sichuan Province, Guangdong Province, Zhejiang Province and Shanghai. The initial batch of samples were 773 *N. gonorrhoea*e isolates derived from isolate repositories corresponding to individuals with symptomatic gonorrhea or urethritis (dysuria and/or urethral discharge). These 773 isolates were collected consecutively between 2019 and 2020, 725 were obtained from male patients, and 48 were obtained from female patients. The *N. gonorrhoeae* isolates consisted of 208 from Zhejiang hospital, 162 from Sichuan hospital, 200 from Guangdong hospital, and 203 from Shanghai hospital.

The second sort of samples consisted of 718 urogenital swabs collected from individuals who sought care for suspected gonorrhoea or urethritis at sexually transmitted infection clinics within the four hospitals between January and July 2021. Urethral swabs were provided by male participants, while cervical swabs were contributed by female participants. All collected secretion swabs were appropriately

stored in the manufacturer's designated specimen transport tubes at room temperature. Among these swabs, 473 (65.88%) originated from male participants, and 245 (34.12%) from female participants. The distribution of secretion swabs was as follows: 104 from Zhejiang hospital, 38 from Sichuan hospital, 207 from Guangdong hospital, and 369 from Shanghai hospital, respectively.

HRM-NG-AMR assay

Nucleic acid extraction was performed using the QIAamp DNA Mini Kit (QIAgen, Hilden, Germany) in accordance with the manufacturer's protocol. A total of 0.2 mL of N. gonorrhoeae bacterial suspension or swab aliquots was processed, and the resultant DNA was eluted in a volume of 100 μL. The DNA preparation phase was diligently executed by the respective hospitals. Subsequently, these DNA samples were preserved at -80℃ and dispatched on dry ice to the IPB (NHC Key Laboratory of Systems Biology of Pathogens, Institute of Pathogen Biology, Beijing, China) for HRM-NG-FC428 testing. The implementation of the HRM-NG-FC428 assay adhered to established protocols [1]. The multiplex HRM reaction was executed through a 20 μL reaction composition, encompassed 10μL of EvaGreen Master Mix (Biotium, Hayward, CA, USA), an optimized quantity of primer mix, 2 μL of the DNA template, and the requisite volume of nuclease-free water to achieve a total volume of 20 μL. The experiments were conducted utilizing a QuantStudio 6 Flex instrument (Applied Biosystems, Foster City, CA, USA) and were facilitated using 96-well PCR plates. The HRM-NG-FC428 assay was conducted under the following conditions: (i) initial denaturation, commencing at 95°C for a duration of 10 minutes. (ii) multiplex PCR thermocycling comprising 35 cycles, with temperature cycles of 95°C for 10 seconds and 60°C for 1 minute. (iii) HRM analysis, which involved an initial holding step of 1 minute at 60°C, succeeded by a gradual temperature rise at a rate of 0.025°C/s reaching 95°Cwhile continuously monitoring fluorescence signals. Each assay incorporated both positive and negative controls. For the three distinct target allele types within the study, *penA*-60.001 was designated as the positive control representing mosaic *penA* alleles harboring the A311V mutation, *penA*-10.001 served as the positive control for mosaic

penA alleles without the A311V mutation, and *penA*-2.001 was selected as the positive control for non-mosaic *penA* alleles. In contrast, ddH2O was employed as the negative control instead of template DNA. The resultant data were analyzed employing the QuantStudio 6 and 7 Flex Real-Time PCR software v1.0 (Applied Biosciences, Foster City, CA, USA). For the interpretation of the results, refer to the standards and guidelines outlined in our previously published study [1].

PCR and bidirectional sequencing analysis

To validate the FC428-related strains identified through the HRM-NG-FC428 assay, complete *penA* gene sequencing was performed on positive strains and clinical specimens. The PCR-based amplification utilized the *penA*_Forward (5'-ATAGAAGCAGCCTGTGTGCC-3') and *penA*_Reverse (5'-GCCAAAGGGCTTAACTTGCT-3') primers. Each PCR reaction comprised the following components: 12.5 μL of 2 × Taq Master Mix (Vazyme Medical Technology, Nanjing, China), 2 μL of each forward and reverse primer, 2 μL of DNA template, and an appropriate volume of nuclease-free water (Thermo Fisher Scientific) to supply to a total volume of 25 μL. The PCR amplification process involved an initial denaturation step at 95°C for 2 minutes, followed by 35 amplification cycles at 95°C for 30 seconds, 60°C for 30 seconds, and 72°C for 2 minutes, culminating in a final elongation step at 72°C for 7 minutes. Sanger sequencing of the resulting PCR products was performed at Tsingke Biotechnology (Beijing, China) using 3730xl DNA Analyzer (Applied Biosystems, Foster City, CA, USA). Subsequently, the generated *penA* consensus sequences were compiled, refined, and compared against reference strain data using the Geneious software (Biomatters Ltd, Auckland, New Zealand).

References

1.Xiu L, Zhang C, Li Y, Wang F, Peng J. High-resolution melting analysis for rapid detection of the internationally spreading ceftriaxone-resistant *Neisseria gonorrhoeae* FC428 clone. J Antimicrob Chemother. 2020 Jan 1;75(1):106-109. doi:

10.1093/jac/dkz395. PMID: 31834402.

Supplementary Results

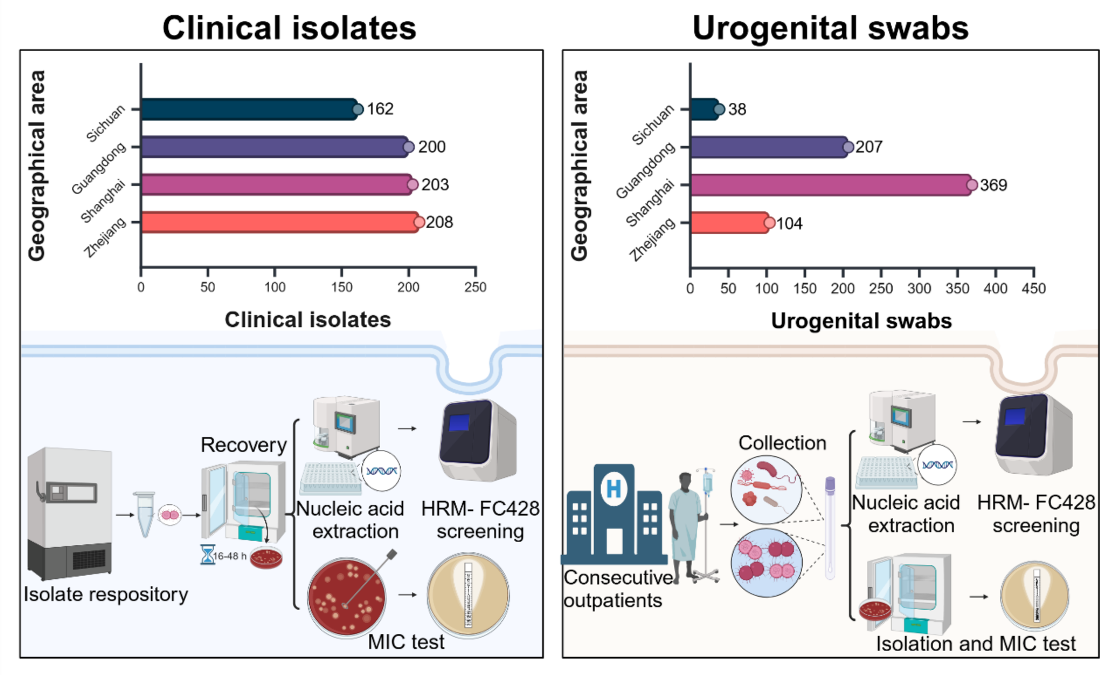

Figure S1 Flowchart of the origin and processing of tested samples used in this study

Table S1 Comparison of phenotypic and molecular characteristics for *N. gonorrhoeae* strain with the novel mosaic *penA* alleles

Characteristic [a]	CD20-24	CD20-63	CD21-8	CD21-24	SH21-335	SH21-355
Province	Sichuan	Sichuan	Sichuan	Sichuan	Shanghai	Shanghai
Year	2020	2020	2021	2021	2021	2021
Gender	Male	Male	Male	Male	Male	Female
Symptom	Gonorrhea	Gonorrhea	Urethritis	Urethritis	Urethritis	Urethritis
Type	Isolate	Isolate	Swab	Swab	Swab	Swab
**MIC (mg/L)**						
CRO	0.25	0.25	0.125	0.125	0.25	0.5
CFM	0.125	0.0625	0.5	1	2	2
SPT	16	16	8	8	8	16
CIP	≥16	≥16	≥32	≥32	16	8
AZM	1	1	≥1	0.5	0.5	8
PEN	1	1	0.5	0.5	0.5	1
**MLST**	7363	7363	7363	7363	13943	7363
abcZ	59	59	59	59	126	59
adk	39	39	39	39	39	39
aroE	67	67	67	67	170	67
fumC	78	78	78	78	157	78
gdh	148	148	148	148	148	148
pdhC	153	153	153	153	153	153
pgm	65	65	65	65	65	65
**NG-MAST**	12697	12697	12697	12697	ND [b]	22283
porB	7342	7342	7342	7342	3238	3237
tbpB	10	10	10	10	ND	206
**NG-STAR**	4510	4510	4510	4510	ND	ND
penA	232.001	232.001	232.001	232.001	195.001	195.001
mtrR	33	33	33	33	1	1
porB	8	8	8	8	ND	8
ponA	1	1	1	1	ND	1
gyrA	2	2	2	2	ND	ND
parC	5	5	5	5	3	ND
23S rRNA	100 (WT)	100	100	100	100	2 (C2611T)

[a] Characteristic: MIC, minimum inhibitory concentration; CRO, ceftriaxone; SPT, spectinomycin; CFM, cefixime; CIP, ciprofloxacin; AZM, azithromycin; PEN, penicillin; MLST: multilocus sequence typing; NG-MAST: *N. gonorrhoeae* multiantigen sequence typing; NG-STAR, *N. gonorrhoeae* sequence typing for antimicrobial resistance;

[b] ND, not described.

Table S2 Target sequences of mosaic *penA* alleles used in sequence alignments

Target alleles	Sequences
penA-37.001	ATGTTGATTAAAAGCGAATATAAGCCCCGGATGCTGCCCAAAGAA<br>GAGCAGGTCAAAAAGCCGATGACCAGTAACGGACGGATTAGCTT<br>CGTCCTGATGGCAATGGCGGTCTTGTTTGCCTGTCTGATTGCCCG<br>CGGGCTGTATCTGCAGACGGTAACGTATAACTTTTTGAAAGAACA<br>GGGCGACAACCGGATTGTGCGGACTCAAGCATTGCCGGCTACAC<br>GCGGTACGGTTTCGGACCGGAACGGTGCGGTTTTGGCGTTGAGC<br>GCGCCGACGGAGTCCCTGTTTGCCGTACCTAAAGAGATGAAGGA<br>AATGCCGTCTGCCGCCCAATTGGAACGCCTTTCCGAGCTTGTCGA<br>TGTGCCGGTTGATGTTTTGAGAAACAAACTCGAACAGAAAGGCA<br>AGTCGTTTATCTGGATTAAGCGGCAGCTCGATCCCAAGGTTGCCG<br>AAGAGGTCAAGGCCTTGGGTTTGGAAAACTTTGCATTTGAAAAA<br>GAATTAAAACGCCATTACCCGATGGGCAGCCTTTTTGCACACGTC<br>ATCGGATTTACCGATATTGACGGCAAAGGTCAGGAAGGTTTGGAG<br>CTTTCGCTTGAAGACAGTTTGCATGCTGGAGAAGGTGCGGAAGT<br>CGTATTGCGGGATCGGGAAGGCAATATTGTGGACAGTTTGGATTC<br>TCCGCGCAATAAAGCCCCGCAAAACGGCAAAGACATTATTCTTTC<br>TCTGGATCAGAGGATTCAGACTTTGGCTTATGAAGAGTTGAATAA<br>GGCGGTCGAATACCATCAGGCAAAAGCCGGAACGGTGGTGGTGT<br>TGGATGCCCGTACTGGGGAAATCCTCGCATTGGTCAATACGCCTG<br>CCTATGAGCCCAACAAACCCGGTCAGGCAGACAGCGAACAGAG<br>GCGTAACCGCGCCGTAACCGACATGATCGAACCCGGTTCTGTCAT<br>GAAGCCGTTTCCCATTGCCAAAGCGCTGGATTCCGGCAAAGTGG<br>ATACGACCGATACATTCAACACCTTGCCTTACAAAATTGGCCCGG<br>CCACCGTACAAGACACCCACGTTTATCCTACTTTGGATGTGCGCG<br>GCATTATGCAAAAATCTTCCAACGTCGGTACCAGTAAACTTTCTG<br>CCATGTTTACGCCTAAAGAAATGTACGATTTCTATCACGATTTAGG<br>TGTGGGCGTGCGCATGCATTCAGGCTTTCCGGGGGAAAGCGCGG<br>GCGTGTTGCGTAATTGGCGGAAATGGCGGCCTATCGAACAGGCG<br>ACGATGTCTTTCGGTTATGGCCTGCAATTAAGCCTGTTGCAATTGG<br>CACGTGCCTATACTGTCTTGACCCATGACGGCGAATTGTTGCCGG<br>TCAGCTTTGAAAAACAAGCAGTTGCCCCTAAAGGCAAACGCGTC<br>ATCAAAGCCTCTACTGCAAAAAAAGTGCGCGAGTTGATGGTTTC<br>CGTTACTGAAGCCGGCGGTAGCGGTATTGCCGGTGCGGTAGATGG<br>TTTCGACGTCGGCGCAAAAACCGGTACGGCGCGTAAGTTGGTTA<br>ACGGTCGTTACGTCGATTACAAACACGTTGCCACTTTCATCGGTT<br>TTGCCCCGGCTAAAAATCCGCGTGTGATTGTGGCGGTAACCATTG<br>ACGAGCCGACTGCAAACGGTTACTACAGCGGCGTAGTGACAGGT<br>CCGGTCTTCAAACAAGTTATGGGCGGTAGCCTGAACATCTTGGGC<br>GTTTCTCCGACCAAACCTCTGACCAATGTTGCAGCCGTCAAAAC<br>ACCGTCTTAA
penA-59.001	ATGTTGATTAAAAGCGAATATAAGCCCCGGATGCTGCCCAAAGAA

GAGCAGGTCAAAAAGCCGATGACCAGTAACGGACGGATTAGCTT
CGTCCTGATGGCAATGGCGGTCTTGTTTGCCTGTCTGATTGCCCG
CGGGCTGTATCTGCAGACGGTAACGTATAACTTTTTGAAAGAACA
GGGCGACAACCGGATTGTGCGGACTCAAGCATTGCCGGCTACAC
GCGGTACGGTTTCGGACCGGAACGGTGCGGTTTTGGCGTTGAGC
GCGCCGACGGAGTCCCTGTTTGCCGTACCTAAAGAGATGAAGGA
AATGCCGTCTGCCGCCCAATTGGAACGCCTTTCCGAGCTTGTCGA
TGTGCCGGTTGATGTTTTGAGAAACAAACTCGAACAGAAAGGCA
AGTCGTTTATCTGGATTAAGCGGCAGCTCGATCCCAAGGTTGCCG
AAGAGGTCAAGGCCTTGGGTTTGGAAAACTTTGCATTTGAAAAA
GAATTAAAACGCCATTACCCGATGGGCAACCTTTTTGCACATGTC
ATCGGATTTACCGATATTGACGGCAAAGGTCAGGAAGGTTTGGAG
CTTTCCCGTGAAGACAGCCTGCGCGGTGAAGATGGTGCGAAAGT
TGTATTACGTGACAACAAAGGCAATATCGTAGACAGCCTCGATTC
TCCACGCAACAGCGTGCCTAAAAACGGCCAAGACATGATTTTGT
CTTTGGATCAGCGTATTCAAACGCTGGCTTATGACGAGTTGAACA
AGGCTGTGGCTTATCACAAAGCCAAAGCGGGTACGGTTGTGGTAT
TGGATGCGCAAACCGGCGAGATTTTGGCGTTGGTCAACAGCCCG
GCTTACGATCCAAATCAGCCCGGCCAAGCCAATAGCGAACAGCG
CCGCAACCGCGCCGTAACCGATATGATTGAACCTGGTTCTGTCAT
GAAGCCGTTTACCATTGCCAAAGCATTGGATTCAGGCAAAGTGG
ATCCAACAGACACATTCAATACCCTGCCTTACAAAATCGGTCCGG
CTACCGTACAAGATACCCACGTTTATCCTACTTTGGATGTGCGCGG
CATTATGCAAAAATCTTCCAACGTCGGTACCAGTAAACTTTCTGC
CATGTTTACGCCTAAAGAAATGTACGATTTCTATCACGATTTAGGT
GTGGGCGTGCGCATGCATTCAGGCTTTCCCGGCGAGACTGCAGG
TTTGTTGAGAAGCTGGCGCAGATGGCAAAAAATCGAACAGGCAA
CCATGTCTTTCGGTTATGGCCTGCAATTAAGCCTGTTGCAATTGGC
ACGTGCCTATACTGTCTTGACCCATGACGGCGAATTGTTGCCGGT
CAGCTTTGAAAAACAAGCAGTTGCCCCTAAAGGCAAACGCGTCA
TCAAAGCCTCTACTGCAAAAAAAGTGCGCGAGTTGATGGTTTCC
GTTACTGAAGCCGGCGGTAGCGGTATTGCCGGTGCGGTAGATGGT
TTCGACGTCGGCGCAAAAACCGGTACGGCGCGTAAGTTGGTTAA
CGGTCGTTACGTCGATTACAAACACGTTGCCACTTTCATCGGTTTT
GCCCCGGCTAAAAATCCGCGTGTGATTGTGGCGGTAACCATTGAC
GAGCCGACTGCAAACGGTTACTACAGCGGCGTAGTGACAGGTCC
GGTCTTCAAACAAGTTATGGGCGGTAGCCTGAACATCTTGGGCGT
TTCTCCGACCAAACCTCTGACCAATGTTGCAGCCGTCAAAACAC
CGTCTTAA

penA-60.001 ATGTTGATTAAAAGCGAATATAAGCCCCGGATGCTGCCCAAAGAA
GAGCAGGTCAAAAAGCCGATGACCAGTAACGGACGGATTAGCTT
CGTCCTGATGGCAATGGCGGTCTTGTTTGCCTGTCTGATTGCCCG
CGGGCTGTATCTGCAGACGGTAACGTATAACTTTTTGAAAGAACA
GGGCGACAACCGGATTGTGCGGACTCAAGCATTGCCGGCTACAC

GCGGTACGGTTTCGGACCGGAACGGTGCGGTTTTGGCGTTGAGC
GCGCCGACGGAGTCCCTGTTTGCCGTGCCTAAAGATATGAAGGA
AATGCCGTCTGCCGCCCAATTGGAACGCCTGTCCGAGCTTGTCGA
TGTGCCGGTCGATGTTTTGAGGAACAAACTCGAACAGAAAGGCA
AGTCGTTTATTTGGATCAAGCGGCAGCTCGATCCCAAGGTTGCCG
AAGAGGTCAAAGCCTTGGGTTTGGAAAACTTTGTATTTGAAAAA
GAATTAAAACGCCATTACCCGATGGGCAACCTGTTTGCACACGTC
ATCGGATTTACCGATATTGACGGCAAAGGTCAGGAAGGTTTGGAA
CTTTCGCTTGAAGACAGCCTGTATGGCGAAGACGGCGCGGAAGT
TGTTTTGCGGGACCGGCAGGGCAATATTGTGGACAGCTTGGACTC
CCCGCGCAATAAAGCACCGCAAAACGGCAAAGACATCATCCTTT
CCCTCGATCAGAGGATTCAGACCTTGGCCTATGAAGAGTTGAACA
AGGCGGTCGAATACCATCAGGCAAAAGCCGGAACGGTGGTGGTT
TTGGATGCCCGCACGGGGGAAATCCTCGCCTTGGCCAATACGCCC
GCCTACGATCCCAACAGACCCGGCCGGGCAGACAGCGAACAGC
GGCGCAACCGTGCCGTTACCGACATGATCGAACCTGGTTCTGTCA
TGAAGCCGTTTACCATTGCCAAAGCATTGGATTCAGGCAAAGTGG
ATCCAACAGACACATTCAATACCCTGCCTTACAAAATCGGTCCGG
CTACCGTACAAGATACCCACGTTTATCCTACTTTGGATGTGCGCGG
CATTATGCAAAAATCTTCCAACGTCGGTACCAGTAAACTTTCTGC
CATGTTTACGCCTAAAGAAATGTACGATTTCTATCACGATTTAGGT
GTGGGCGTGCGCATGCATTCAGGCTTTCCCGGCGAGACTGCAGG
TTTGTTGAGAAGCTGGCGCAGATGGCAAAAAATCGAACAGGCAA
CCATGTCTTTCGGTTATGGCCTGCAATTAAGCCTGTTGCAATTGGC
GCGTGCCTATACTGTCTTGACCCATGACGGCGAATTGTTGCCGGT
CAGCTTTGAAAAACAAGCAGTTGCCCCTAAAGGCAAACGCGTCA
TCAAAGCCTCTACTGCAAAAAAAGTGCGCGAGTTGATGGTTTCC
GTTACTGAAGCCGGCGGTAGCGGTATTGCCGGTGCGGTAGATGGT
TTCGACGTCGGCGCAAAAACCGGTACGGCGCGTAAGTTGGTTAA
CGGTCGTTACGTCGATTACAAACACGTTGCCACTTTCATCGGTTTT
GCCCCGGCTAAAAATCCGCGTGTGATTGTGGCGGTAACCATTGAC
GAGCCGACTGCAAACGGTTACTACAGCGGCGTAGTGACAGGTCC
GGTCTTCAAACAAGTTATGGGCGGTAGCCTGAACATCTTGGGCGT
TTCTCCGACCAAACCTCTGACCAATGTTGCAGCCGTCAAAACAC
CGTCTTAA

penA-64.001 ATGTTGATTAAAAGCGAATATAAGCCCCGGATGCTGCCCAAAGAA
GAGCAGGTCAAAAAGCCGATGACCAGTAACGGACGGATTAGCTT
CGTCCTGATGGCAATGGCGGTCTTGTTTGCCTGTCTGATTGCCCG
CGGGCTGTATCTGCAGACGGTAACGTATAACTTTTTGAAAGAACA
GGGCGACAACCGGATTGTGCGGACTCAAGCATTGCCGGCTACAC
GCGGTACGGTTTCGGACCGGAACGGTGCGGTTTTGGCGTTGAGC
GCGCCGACGGAGTCCCTGTTTGCCGTACCTAAAGAGATGAAGGA
AATGCCGTCTGCCGCCCAATTGGAACGCCTTTCCGAGCTTGTCGA
TGTGCCGGTTGATGTTTTGAGAAACAAACTCGAACAGAAAGGCA

AGTCGTTTATCTGGATTAAGCGGCAGCTCGATCCCAAGGTTGCCG
AAGAGGTCAAGGCCTTGGGTTTGGAAAACTTTGCATTTGAAAAA
GAATTAAAACGCCATTACCCGATGGGCAGCCTTTTTGCACACGTC
ATCGGATTTACCGATATTGACGGCAAAGGTCAGGAAGGTTTGGAA
CTTTCGCTTGAAGACAGTTTGCATGCTGGAGAAGGTGCGGAAGT
CGTATTGCGGGATCGGGAAGGCAATATTGTGGACAGTTTGGATTC
TCCGCGTAATAAAGCTCCGCAAAACGGCAAAGACATTATTCTTTC
TCTGGATCAGAGGATTCAGACTTTGGCTTATGAAGAGTTGAACAA
GGCGGTTGAATACCATCAGGCAAAAGCCGGCACGGTGGTGGTTT
TGGATGCCCGCACGGGGGAAATCCTCGCCTTGGCCAACACGCCC
GCCTACGATCCCAACAGACCCGGCCGGGCAGACAGCGAACAGA
GGCGCAACCGCGCCGTAACCGACATGATCGAACCTGGTTCTGTC
ATGAAGCCGTTTACCATTGCCAAAGCATTGGATTCAGGCAAAGTG
GATCCAACAGACACATTCAATACCCTGCCTTACAAAATCGGTCCG
GCTACCGTACAAGATACCCACGTTTATCCTACTTTGGATGTGCGCG
GCATTATGCAAAAATCTTCCAACGTCGGTACCAGTAAACTTTCTG
CCATGTTTACGCCTAAAGAAATGTACGATTTCTATCACGATTTAGG
TGTGGGCGTGCGCATGCATTCAGGCTTTCCCGGCGAGACTGCAG
GTTTGTTGAGAAGCTGGCGCAGATGGCAAAAAATCGAACAGGCA
ACCATGTCTTTCGGTTATGGCCTGCAATTAAGCCTGTTGCAATTGG
CACGTGCCTATACTGTCTTGACCCATGACGGCGAATTGTTGCCGG
TCAGCTTTGAAAAACAAGCAGTTGCCCCTAAAGGCAAACGCGTC
ATCAAAGCCTCTACTGCAAAAAAAGTGCGCGAGTTGATGGTTTC
CGTTACTGAAGCCGGCGGTAGCGGTATTGCCGGTGCGGTAGATGG
TTTCGACGTCGGCGCAAAAACCGGTACGGCGCGTAAGTTGGTTA
ACGGTCGTTACGTCGATTACAAACACGTTGCCACTTTCATCGGTT
TTGCCCCGGCTAAAAATCCGCGTGTGATTGTGGCGGTAACCATTG
ACGAGCCGACTGCAAACGGTTACTACAGCGGCGTAGTGACAGGT
CCGGTCTTCAAACAAGTTATGGGCGGTAGCCTGAACATCTTGGGC
GTTTCTCCGACCAAACCTCTGACCAATGTTGCAGCCGTCAAAAC
ACCGTCTTAA

penA-195.001

ATGTTGATTAAAAGCGAATATAAGCCCCGGATGCTGCCCAAAGAA
GAGCAGGTCAAAAAGCCGATGACCAGTAACGGACGGATTAGCTT
CGTCCTGATGGCAATGGCGGTCTTGTTTGCCTGTCTGATTGCCCG
CGGGCTGTATCTGCAGACGGTAACGTATAACTTTTTGAAAGAACA
GGGCGACAACCGGATTGTGCGGACTCAAGCATTGCCGGCTACAC
GCGGTACGGTTTCGGACCGGAACGGTGCGGTTTTGGCGTTGAGC
GCGCCGACGGAGTCCCTGTTTGCCGTACCTAAAGAGATGAAGGA
AATGCCGTCTGCCGCCCAATTGGAACGCCTTTCCGAGCTTGTCGA
TGTGCCGGTTGATGTTTTGAGAAACAAACTCGAACAGAAAGGCA
AGTCGTTTATCTGGATTAAGCGGCAGCTCGATCCCAAGGTTGCCG
AAGAGGTCAAGGCCTTGGGTTTGGAAAACTTTGCATTTGAAAAA
GAATTAAAACGCCATTACCCGATGGGCAGCCTTTTTGCACACGTC
ATCGGATTTACCGATATTGACGGCAAAGGTCAGGAAGGTTTGGAG

	CTTTCGCTTGAAGACAGTTTGCATGCTGGAGAAGGTGCGGAAGT<br>CGTATTGCGGGATCGGGAAGGCAATATTGTGGACAGTTTGGATTC<br>TCCGCGCAATAAAGCCCCGCAAAACGGCAAAGACATTATTCTTTC<br>TCTGGATCAGAGGATTCAGACTTTGGCTTATGAAGAGTTGAATAA<br>GGCGGTCGAATACCATCAGGCAAAAGCCGGAACGGTGGTGGTGT<br>TGGATGCCCGTACTGGGGAAATCCTCGCATTGGTCAATACGCCTG<br>CCTATGAGCCCAACAAACCCGGTCAGGCAGACAGCGAACAGAG<br>GCGTAACCGCGCCGTAACCGACATGATCGAACCCGGTTCTGTCAT<br>GAAGCCGTTTACCATTGCCAAAGCATTGGATTCCGGCAAAGTGG<br>ATGCAACCGATACATTCAATACCCTGCCTTACAAAATCGGTTCGG<br>CTACCGTACAAGATACCCACGTTTATCCTACTTTGGATGTGCGCGG<br>CATTATGCAAAAATCTTCCAACGTCGGTACCAGTAAACTTTCTGC<br>CATGTTTACGCCTAAAGAAATGTACGATTTCTATCACGATTTAGGT<br>GTGGGCGTGCGCATGCATTCAGGCTTTCCTGGTGAAACTGCAGGT<br>TTGTTGAGAAGCTGGCGCAGATGGCAAAAAATCGAACAGGCAAC<br>CATGTCTTTCGGTTATGGCCTGCAATTAAGCCTGTTGCAATTGGCG<br>CGTGCCTATACTGTCTTGACCCATGACGGCGAATTGTTGCCGGTC<br>AGCTTTGAAAAACAAGCGGTTGCGCCTAAAGGCAAGCGCGTCAT<br>CAAAGCCTCTACTGCCAAAAAAGTGCGCGAGTTGATGGTTTCCG<br>TTACTGAAGCCGGCGGTACCGGTACTGCTGGTGCGGTAGATGGTT<br>TCGACGTCGGCGCAAAAACCGGTACGGCGCGTAAGTTGGTTAAC<br>GGTCGTTACGTCGATTACAAACACGTTGCCACTTTCATCGGTTTT<br>GCCCCGGCTAAAAATCCGCGTGTGATTGTGGCGGTAACCATTGAC<br>GAGTCGACTGCAAACGGTTACTACAGCGGTGTAGTGACAGGTCC<br>GGTCTTCAAACAAGTGATGGGCGGCAGCCTGAACATCTTGGGCG<br>TTTCCCCGACCAAACCTTTGACCAATGTTGCAGCCGTCAAAACAC<br>CGTCTTAA
penA-232.001	ATGTTGATTAAAAGCGAATATAAGCCCCGGATGCTGCCCAAAGAA<br>GAGCAGGTCAAAAAGCCGATGACCAGTAACGGACGGATTAGCTT<br>CGTCCTGATGGCAATGGCGGTCTTGTTTGCCTGTCTGATTGCCCG<br>CGGGCTGTATCTGCAGACGGTAACGTATAACTTTTTGAAAGAACA<br>GGGCGACAACCGGATTGTGCGGACTCAAGCATTGCCGGCTACAC<br>GCGGTACGGTTTCGGACCGGAACGGTGCGGTTTTGGCGTTGAGC<br>GCGCCGACGGAGTCCCTGTTTGCCGTACCTAAAGAGATGAAGGA<br>AATGCCGTCTGCCGCCCAATTGGAACGCCTTTCCGAGCTTGTCGA<br>TGTGCCGGTTGATGTTTTGAGAAACAAACTCGAACAGAAAGGCA<br>AGTCGTTTATCTGGATTAAGCGGCAGCTCGATCCCAAGGTTGCCG<br>AAGAGGTCAAGGCCTTGGGTTTGGAAAACTTTGCATTTGAAAAA<br>GAATTAAAACGCCATTACCCGATGGGCAGCCTTTTTGCACACGTC<br>ATCGGATTTACCGATATTGACGGCAAAGGTCAGGAAGGTTTGGAG<br>CTTTCGCTTGAAGACAGTTTGCATGCTGGAGAAGGTGCGGAAGT<br>CGTATTGCGGGATCGGGAAGGCAATATTGTGGACAGTTTGGATTC<br>TCCGCGCAATAAAGCCCCGCAAAACGGCAAAGACATTATTCTTTC<br>TCTGGATCAGAGGATTCAGACTTTGGCTTATGAAGAGTTGAATAA

GGCGGTCGAATACCATCAGGCAAAAGCCGGAACGGTGGTGGTGT
TGGATGCCCGTACTGGGGAAATCCTTGCCTTGGTCAATACGCCTG
CCTATGAGCCCAACAAACCCGGTCAGGCAGACAGCGAACAGCG
GCGCAACCGCGCCGTAACCGATATGATTGAACCTGGTTCTGTCAT
GAAGCCGTTTACCATTGCCAAAGCATTGGATTCAGGCAAAGTGG
ATCCAACAGACACATTCAATACCCTGCCTTACAAAATCGGTCCGG
CTACCGTACAAGATACCCACGTTTATCCTACTTTGGATGTGCGCGG
CATTATGCAAAAATCTTCCAACGTCGGTACCAGTAAACTTTCTGC
CATGTTTACGCCTAAAGAAATGTACGATTTCTATCACGATTTAGGT
GTGGGCGTGCGCATGCATTCAGGCTTTCCCGGCGAGACTGCAGG
TTTGTTGAGAAGCTGGCGCAGATGGCAAAAAATCGAACAGGCAA
CCATGTCTTTCGGTTATGGCCTGCAATTAAGCCTGTTGCAATTGGC
ACGTGCCTATACTGTCTTGACCCATGACGGCGAATTGTTGCCGGT
CAGCTTTGAAAAACAAGCAGTTGCCCCTAAAGGCAAACGCGTCA
TCAAAGCCTCTACTGCAAAAAAAGTGCGCGAGTTGATGGTTTCC
GTTACTGAAGCCGGCGGTAGCGGTATTGCCGGTGCGGTAGATGGT
TTCGACGTCGGCGCAAAAACCGGTACGGCGCGTAAGTTGGTTAA
CGGTCGTTACGTCGATTACAAACACGTTGCCACTTTCATTGGTTTT
GCCCCGGCTAAAAATCCGCGTGTGATTGTGGCGGTAACCATTGAC
GAGCCGACTGCAAACGGTTACTACAGCGGCGTAGTGACAGGTCC
GGTCTTCAAACAAGTTATGGGCGGTAGCCTGAACATCTTGGGCGT
TTCTCCGACCAAACCTCTGACCAATGTTGCAGCCGTCAAAACAC
CGTCTTAA
